# Mortality from Unspecified Unintentional Injury among Individuals Aged 65 Years and Older by U.S. State, 1999–2013

**DOI:** 10.3390/ijerph13080763

**Published:** 2016-07-27

**Authors:** Xunjie Cheng, Yue Wu, Jie Yao, David C. Schwebel, Guoqing Hu

**Affiliations:** 1 Department of Epidemiology and Health Statistics, Xiangya School of Public Health, Central South University, 110 Xiangya Road, Changsha 410078, Hunan, China; chengxunjie163@163.com; 2 Department of Occupational and Environmental Health, Xiangya School of Public Health, Central South University, 110 Xiangya Road, Changsha 410078, Hunan, China; wuyue7802@csu.edu.cn; 3 College of Humanities and Social Sciences, Harbin Institute of Technology (Shenzhen), Xili, Nanshan District, Shenzhen 518055, Guangdong, China; yaojie@hitsz.edu.cn; 4 Department of Psychology, University of Alabama at Birmingham, Birmingham, AL 35294, USA; schwebel@uab.edu

**Keywords:** unintentional injury, mortality, specificity, elderly, U.S.

## Abstract

Introduction: Recent changes in unspecified unintentional injury mortality for the elderly by U.S. state remain unreported. This study aims to examine U.S. state variations in mortality from unspecified unintentional injury among Americans aged 65+, 1999–2013; Methods: Using mortality rates from the U.S. CDC’s Web-based Injury Statistics Query and Reporting System (WISQARS™), we examined unspecified unintentional injury mortality for older adults aged 65+ from 1999 to 2013 by state. Specifically, the proportion of unintentional injury deaths with unspecified external cause in the data was considered. Linear regression examined the statistical significance of changes in proportion of unspecified unintentional injury from 1999 to 2013; Results: Of the 36 U.S. states with stable mortality rates, over 8-fold differences were observed for both the mortality rates and the proportions of unspecified unintentional injury for Americans aged 65+ during 1999–2013. Twenty-nine of the 36 states showed reductions in the proportion of unspecified unintentional injury cause, with Oklahoma (−89%), Massachusetts (−86%) and Oregon (−81%) displaying the largest changes. As unspecified unintentional injury mortality decreased, mortality from falls in 28 states and poisoning in 3 states increased significantly. Mortality from suffocation in 15 states, motor vehicle traffic crashes in 12 states, and fire/burn in 8 states also decreased; Conclusions: The proportion of unintentional injuries among older adults with unspecified cause decreased significantly for many states in the United States from 1999 to 2013. The reduced proportion of unspecified injury has implications for research and practice. It should be considered in state-level trend analysis during 1999–2013. It also suggests comparisons between states for specific injury mortality should be conducted with caution, as large differences in unspecified injury mortality across states and over time could create bias for specified injury mortality comparisons.

## 1. Introduction

The proportion of injuries reported with an unspecified cause of injury can substantially affect the reported morbidity and mortality rates of cause-specific injury data. Researchers can approximate this proportion using the proportion of injury deaths with unspecified external cause codes [1,2,3], and some researchers suggest a proportion larger than about 10% [2] or 20% [3] of total deaths assigned to ill-defined or non-specific codes is considered “poor quality” or “unsatisfactory”. The National Center for Health Statistics (NCHS) within the U.S. Centers for Disease Control and Prevention has conducted special studies and expanded outreach to physicians and others completing birth and death certificates, with a goal to improve data quality and reduce the proportion of injuries coded with an unspecified cause of injury [4]. Much of this work has focused on older adults aged 65+, where a high proportion of errors occur, and two studies suggest these efforts have been successful over the last two decades at the national level [5,6]. Stevens and Rudd reported a trend toward more-specific reporting of circumstances for falls for Americans aged 65 years and older from 1999 to 2010, although total deaths remained unchanged [5]. Similarly, Hu and Mamady reported that the proportion of unspecified unintentional injuries decreased from 18.9% to 10.9% between 1999 and 2010 for the elderly aged 65+ at the national level, and that the decrease in unspecified unintentional injury mortality was correlated with changes in mortality rates from motor vehicle crashes, falls, poisonings, drownings, suffocations, fires/burns, and natural/environmental disasters [6]. These results suggest that efforts to improve specificity in reporting the cause of injury mortality may have resulted in improved data quality and more accurate tallying of the causes of injuries among older adults nationally.

Both previous studies were conducted at the national level, however. Most injury reporting and analysis are conducted locally, and there are large variations across U.S. states in coding practices [7,8,9,10,11,12]. Therefore data analysis at the state level is needed and could reveal state-by-state differences in the proportion of unspecified unintentional injury deaths among older American adults that have implications for policy-makers, researchers and others. The current research examined changes in the proportion of unspecified unintentional injury mortality rates among Americans aged 65+ at the state level. We also calculated Spearman rank correlations between unspecified unintentional injury mortality and five major cause-specific injury mortality rates for the elderly at the state level. Analyses were conducted using data from 1999 to 2013.

## 2. Materials

### 2.1. Data Source

Mortality data were obtained through the U.S. CDC’s Web-based Injury Statistics Query and Reporting System (WISQARS™), which provides fatal and nonfatal injury, violent death, and cost of injury data [13]. WISQARS Fatal Injury Data provide the total numbers of injury-related deaths and death rates per 100,000 population by cause (mechanism) and intent (manner) of injury, state of residence, race, Hispanic origin, sex, and age [13]. Data are based on annual mortality data compiled by the National Center for Health Statistics (NCHS) at CDC. Since 1999, the cause of deaths has been coded using the 10th International Classification of Diseases (ICD-10). We therefore began our analysis with data from 1999. We used 2013 for the final year to analyze trends in the data.

### 2.2. Statistical Analysis

Adapting techniques used in previous research [1,6], we used the proportion of unintentional injury deaths coded with an unspecified cause to estimate the precision in coding injury data for the cause of injury. Our analyses proceeded in three steps. 

First, we described data concerning injury mortality rate not being given a specific cause in each U.S. state in both 1999 and 2013. We excluded states with unstable mortality rates from the above analyses since mortality rates based on fewer than 20 deaths are unstable and are not recommended by the CDC WISQARS™ [6]. Based on the extent of change in proportion of unspecified injury mortality at the state level, we divided states with stable mortality rates into four categories: (1) states with ≥60% increase; (2) states with 40%–59% increase; (3) states with 16%–39% increase, and states without significant change. Graphs were prepared to illustrate the trends over time in the proportion of unspecified unintentional injury mortality among each of these four categories of states, as well as for the whole country.

Second, we examined the proportion of unintentional injury deaths coded with unspecified cause from 1999 to 2013 in each U.S. state. Following the strategies of Hu and Baker [14], we used the percent change in proportion to quantify change between 1999 and 2013, which was calculated as “(regression coefficient *14*100%)/the proportion in 1999”. Linear regression models examined the statistical significance of trends in the change from 1999 to 2013.

Last, Spearman rank correlation analysis examined relations between unspecified unintentional mortality rates and cause-specific mortality rates of five major injury causes (falls, motor vehicle traffic crashes, suffocations, fires/burns, poisonings) from 1999 to 2013. “*p* < 0.05” was considered statistically significant. To explore the possibility of compensatory changes between specified injury mortality and unspecified injury mortality, we conducted Spearman rank correlation analysis for states not showing significant reductions in the proportion of unintentional injury code with unspecific cause in addition to those displaying significant reductions. 

## 3. Results

Fourteen U.S. states plus the District of Columbia, all with comparatively small populations, were excluded from analysis because they lacked stable mortality rates: Alaska, Delaware, Hawaii, Idaho, Maine, Montana, Nevada, New Hampshire, New Mexico, North Dakota, Rhode Island, South Dakota, Vermont, and Wyoming.

Among the 36 states with stable mortality rates, over 8-fold differences in rates of unspecified unintentional injuries among individuals ages 65 and over were observed between states in 1999 (i.e., Minnesota: 36.8 per 100,000 persons vs. Wisconsin: 4.1 per 100,000 persons) and in 2013 (i.e., Utah: 26.6 per 100,000 persons vs. Arizona: 2.8 per 100,000 persons) (Table 1). Similar gaps between states were observed for the proportion of those injuries coded with unspecified cause in 1999 (Massachusetts: 36% vs. Wisconsin: 4%) and in 2013 (Alabama: 27% vs. Arizona: 2%).

**Figure 1:**
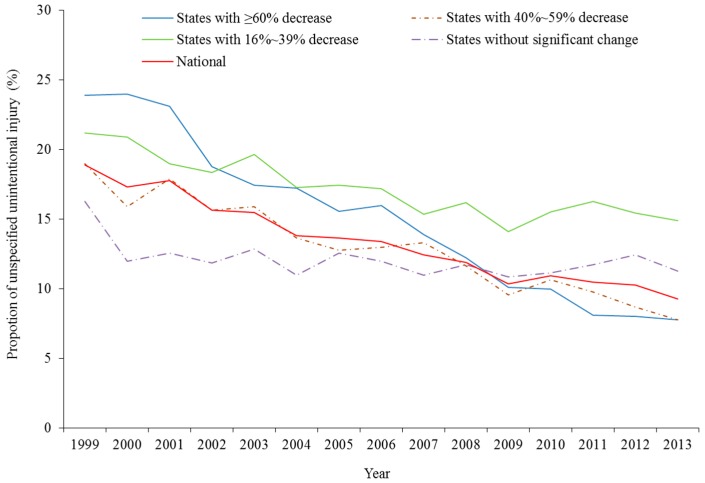
Proportion of unspecified unintentional injury between 1999 and 2013 among Americans aged 65+ by the extent of change. Note: (1) States with ≥60% decrease: Oklahoma, Massachusetts, Oregon, Iowa, Minnesota, Illinois, Arizona, Ohio, Michigan; (2) States with 40%–59% decrease: Colorado, Pennsylvania, Louisiana, North Carolina, Maryland, West Virginia, New York, Texas, California; (3) States with 16%–39% decrease: Virginia, Connecticut, Missouri, Arkansas, Indiana, New Jersey, Florida, Mississippi, Kentucky, Kansas, Alabama; and (4) States without significant change: Washington, Wisconsin, Georgia, South Carolina, Utah, Tennessee, Nebraska.

Between 1999 and 2013, 29 of the 36 states experienced significant reductions in the proportion of unspecified unintentional injury (Table 1). The largest reductions were seen in Oklahoma (−89%), Massachusetts (−86%), Oregon (−81%), Iowa (−75%), Minnesota (−68%), Arizona (−67%), Illinois (−67%), Michigan (−61%), and Ohio (−61%) (Table 1, Figure 1). Another 20 states displayed moderate reductions in the proportion of unspecified unintentional injury. In contrast, the proportion of unspecified unintentional injury did not significantly change for seven states from 1999 to 2013: Washington, Wisconsin, Georgia, South Carolina, Utah, Tennessee, and Nebraska.

Spearman rank correlation found that among the 29 states that experienced distinct reductions in the proportion of unspecified unintentional injury, 28 displayed large increases in fall mortality as the proportion of unspecified unintentional injury dropped from 1999 to 2013 (*r_s_* range from −0.98 to −0.56) (Table 2). As the proportion of unspecified unintentional injury decreased, significant increases in poisoning mortality were also observed in California, Michigan and New York. Finally, we detected significant reductions in mortality rates from unintentional suffocations, motor vehicle traffic crashes, and fires/burns in 15 states, 12 states, and 8 states, respectively as the proportion of unintentional injuries with unspecified cause dropped from 1999 to 2013 (Table 2).

Among the seven U.S. states that did not experience a significant change in the proportion of unintentional injuries to older adults attributed to unspecified causes, most associations between unspecified unintentional injury mortality and specific injury mortality were not statistically significant (Table 2). In fact, only two moderate correlations emerged among those seven states: associations between fall mortality and unintentional injury deaths with unspecified cause in Nebraska (*r_s_* = −0.63) and South Carolina (*r_s_* = −0.52).

## 4. Discussion

We found that (a) 81% of U.S. states with stable mortality rates witnessed reductions in the proportion of unspecified unintentional injury mortality among Americans aged 65+ from 1999 to 2013; (b) changes in the proportion of unspecified unintentional injury mortality between 1999 and 2013 varied greatly across states—Oklahoma, Massachusetts, and Oregon decreased by over 80% but another seven states did not change significantly; and (c) the decreases in rates of unintentional injury mortality with unspecified cause were accompanied with increased mortality from falls in 28 states and from poisoning in 3 states, but decreases in mortality from suffocation in 15 states, motor vehicle traffic crashes in 12 states, and fire/burn in 8 states.

Our findings provide evidence that, consistent with CDC goals [4], the specificity of older adult unintentional injury mortality cause data has improved in 81% of the U.S. states with stable mortality rates (i.e., 29 out of 36 states) [5,15,16]. Notably, however, large differences emerged across states, with some having very significant changes and others showing little or no improvement. It is difficult to speculate precisely what caused change in some states and not in others, but we hypothesize that it may be the result of a variety of factors, including: (a) the quality of training and practice for death coders in hospitals or by coroners and medical examiners; (b) the research capacity and investment to mortality statistics in individual states; and/or (c) the baseline level of unspecified injury mortality causes in 1999. Continued efforts and resources devoted to high-quality collection of injury mortality statistics could help state health departments identify problems in data quality and develop solutions to promote data quality. Further studies are needed also to explore the reasons leading to large reductions of proportion of unintentional injury coded with unspecified cause in some U.S. states and to generalize the successful methods to other U.S. states (as well as globally, as many of these same coding challenges emerge worldwide).

We observed correlations between decreases in unintentional injury mortality with unspecified causes and increases or decreases in cause-specific injury mortality within some states. Such results are consistent with previously-reported findings at the national level [6]. Significant increases in cause-specific injury mortality that we detected, especially in falls and poisoning, may reflect a compensatory result of improved coding practices to some extent. Further, the negative correlations between fall mortality and unintentional mortality with unspecified cause in 28 states suggest that perhaps the recently reported increases in elderly fall mortality in many states [13] may be overestimated to some extent because it reflects not only a real increase but also the effect of a reduced proportion of unspecified unintentional injury mortality.

Our finding that injury mortality with unspecified cause was associated with decreases in mortality from road traffic crashes and suffocation in some states is difficult to explain. It may reflect the impact of factors outside coding issues such as economic recession and injury prevention efforts. Economic recession may influence the choice of transportation method and create safer road usage [17,18]. Reductions in mortality from suffocation may also be the effect of local prevention efforts. Further analysis is needed to explore the reasons behind mortality reductions.

Our findings have three major implications. First, large variations across U.S. states in the proportion of unspecified injury mortality data and in its changes over the years merit further attention from researchers and state health policy-makers in the United States. Federal and U.S. state governments should support research to explore the reasons for state variations and especially to develop methods that help states with relatively high proportions of unspecified injury mortality improve their data quality. Second, the impact of proportion of unspecified injury of data should be considered when examining trends in cause-specific mortality at the state level. This is particularly true for strong correlations between unspecified unintentional injury mortality and cause-specific mortality, as we found for elderly falls. Significant negative or positive correlations between unspecified unintentional mortality and cause-specific mortality indicate that the cause-specific mortality rates are likely to be overestimated or underestimated to some extent [6]. For the same reason, comparisons of cause-specific mortality between states should be interpreted with caution. Third, our findings have implications that may extend beyond the United States since data coding issues occur in other countries also [19]. Policy implications that emerge in individual U.S. states are similarly relevant in individual states/provinces and countries with similar changes (either improvement or deterioration) in mortality data quality. For example, the rate of undetermined deaths decreased from 6.9 per 100,000 to 5.7 per 100,000 in Japan and from 2.8 per 100,000 to 0.3 per 100,000 in Hong Kong between 1992 and 2011 [19]. The decreasing proportion of undetermined deaths in those jurisdictions may influence apparent trends in specific unintentional injury and intentional injury rates there.

A limitation of this study is the lack of external and detailed death information to validate the accuracy of recorded injury causes. Because of this, we cannot systematically assess the quality of injury mortality data between 1999 and 2013 for each state, identify reasons behind the reducing unspecified unintentional injury mortality among old adults aged 65+, or quantitatively estimate the impact of data quality change on cause-specific injury mortality. Another limitation is the fact that we do not know exactly what changes occurred in how cause of injury death was classified in each individual state; knowledge about these details would impact understanding of the cause of the associations we discovered.

## 5. Conclusions

The proportion of unspecified cause of elderly unintentional injury mortality improved for 81% of U.S. states with stable mortality rates from 1999 to 2013. Proportion of unspecified injury cause should be considered when examining yearly changes in cause-specific injury mortality or comparing cause-specific injury mortality between states. To improve the accuracy of cause-specific injury mortality, it is important to study the quality of data and develop methods to adjust its influence on cause-specific injury mortality. Further quality assessment should not be limited to proportion of unspecified injury cause but should also include attributes of data quality such as completeness, sensitivity, specificity, representativeness, positive predictive value, and positive likelihood ratio [20].

## Figures and Tables

**Table 1:** Mortality rates and proportions of unintentional injury among Americans aged 65+ with unspecified cause by U.S. state, 1999–2013.

State	Mortality Rate		Proportion of Unspecified Unintentional Injury			
	1999	2013	1999	2013	*R*^2^^†^	Percent Change in Proportion ^‡^
Alabama	36.5	25.3	29	27	0.24	−16 *
Arizona	8.4	2.8	8	2	0.77	−67 **
Arkansas	21.5	11.9	19	11	0.24	−35 *
California	4.7	3.7	7	6	0.73	−43 **
Colorado	33.7	11.4	27	7	0.82	−59 **
Connecticut	19.5	15.4	24	15	0.46	−36 **
Florida	4.3	3.9	6	4	0.36	−27 *
Georgia	12.6	8.6	10	9	0.16	−17
Illinois	19.5	10.2	23	12	0.80	−67 **
Indiana	31.0	22.8	28	24	0.56	−33 **
Iowa	17.6	15.1	17	11	0.53	−75 **
Kansas	13.2	11.2	12	8	0.21	−20 *
Kentucky	35.3	25.2	31	22	0.47	−20 **
Louisiana	23.4	12.3	23	13	0.59	−59 **
Maryland	15.5	3.7	19	4	0.48	−51 **
Massachusetts	26.1	8.9	36	11	0.94	−86 **
Michigan	21.8	7.9	25	8	0.92	−61 **
Minnesota	36.8	8.4	27	5	0.91	−68 **
Mississippi	11.5	7.0	9	5	0.23	−25 *
Missouri	27.0	13.2	22	12	0.29	−36 *
Nebraska	15.7	13.5	14	12	−0.04	−11
New Jersey	20.0	17.3	26	23	0.39	−29 **
New York	10.4	5.0	15	7	0.83	−51 **
North Carolina	24.9	11.8	21	10	0.73	−59 **
Ohio	27.3	10.4	30	10	0.95	−61 **
Oklahoma	23.0	10.9	19	7	0.63	−89 **
Oregon	30.9	5.7	30	4	0.65	−81 **
Pennsylvania	22.6	9.1	24	9	0.86	−59 **
South Carolina	22.4	15.8	17	14	0.06	−15
Tennessee	33.3	23.7	28	17	0.00	−12
Texas	18.3	7.8	19	8	0.56	−44 **
Utah	32.4	26.6	35	19	0.05	−13
Virginia	27.9	14.8	27	13	0.67	−36 **
Washington	4.8	5.7	6	5	0.07	−21
West Virginia	16.9	8.1	16	6	0.48	−51 **
Wisconsin	4.1	5.4	4	3	−0.01	−20

* *p* < 0.05; ** *p* < 0.01; ^†^ The determination coefficient of linear regression (*y* = proportion of unspecified unintentional injury, *x* = year); ^‡^ Percent change in proportion was calculated as ‘regression coefficient *14*100% divided by the proportion in 1999’. Note: states with unstable mortality rates that were based on 20 or fewer deaths were excluded from analysis.

**Table 2:** Spearman correlation coefficients between unintentional injury mortality among individuals age 65 and over with unspecified cause and injury mortality from particular causes by state in the United States, 1999–2013.

State	*r*_*s*-falls_	*r*_*s*-suffocation_	*r*_*s*-MVT_	*r*_*s*-fire/burn_	*r*_*s*-poisoning_
Alabama **^a^**	−0.65 **	0.14	0.17	0.68 **	―
Arizona **^a^**	−0.91 **	0.65 **	―	―	―
Arkansas **^a^**	−0.56 *	−0.46	−0.13	―	―
California **^a^**	−0.88 **	0.38	0.78 **	0.80 **	−0.86 **
Colorado **^a^**	−0.88 **	0.38	0.48	―	―
Connecticut **^a^**	−0.80 **	0.63 *	0.53 *	―	―
Florida **^a^**	−0.49	0.53 *	0.34	0.43	−0.23
Illinois **^a^**	−0.90 **	−0.17	0.86 **	0.58 *	−0.39
Indiana **^a^**	−0.75 **	0.19	0.63 *	0.05	―
Iowa **^a^**	−0.78 **	0.64 *	0.34	―	―
Kansas **^a^**	−0.70 **	0.65 *	0.33	―	―
Kentucky **^a^**	−0.81 **	0.20	0.45	―	―
Louisiana **^a^**	−0.45	0.48	0.47	0.82 **	―
Maryland **^a^**	−0.71 **	0.55 *	0.46	―	―
Massachusetts **^a^**	−0.98 **	0.66 *	0.66 *	―	―
Michigan **^a^**	−0.98 **	0.68 *	0.55 *	0.10	−0.59 *
Minnesota **^a^**	−0.98 **	−0.25	0.78 **	―	―
Mississippi **^a^**	−0.50	−0.15	0.02	0.33	―
Missouri **^a^**	−0.78 **	0.76 **	0.28	−0.25	―
New Jersey **^a^**	−0.61 *	−0.76 **	0.50	0.35	−0.47
New York **^a^**	−0.93 **	0.21	0.87 **	0.79 **	−0.74 **
North Carolina **^a^**	−0.89 **	0.68 *	0.48	0.45	0.11
Ohio **^a^**	−0.97 **	0.53 *	0.58 *	0.59 *	−0.49
Oklahoma **^a^**	−0.75 **	0.69 **	0.65 *	0.07	―
Oregon **^a^**	−0.97 **	0.31	0.81 **	―	―
Pennsylvania **^a^**	−0.95 **	0.81 **	0.51	0.75 **	−0.47
Texas **^a^**	−0.75 **	0.55 *	0.56	0.45	−0.50
Virginia **^a^**	−0.88 **	0.53 *	0.72 **	0.64 *	―
West Virginia **^a^**	−0.76 **	0.23	0.15	―	―
Georgia **^b^**	−0.46	0.07	0.05	0.18	−0.19
Nebraska **^b^**	−0.63 *	0.35	−0.10	―	―
South Carolina **^b^**	−0.52 *	0.20	0.12	0.20	―
Tennessee **^b^**	−0.25	−0.09	−0.01	0.06	0.19
Utah **^b^**	−0.21	―	−0.11	―	―
Washington **^b^**	−0.49	−0.11	0.33	―	―
Wisconsin **^b^**	0.29	0.37	0.15	―	―

**^a^** State with significant change in proportion of unintentional injury from 1999 to 2013; **^b^** State without significant change in proportion of unintentional injury from 1999 to 2013; * *p* < 0.05; ** *p* < 0.01; ―, States with unstable mortality rates that were based on 20 or fewer deaths were excluded from Spearman correlation analysis; *r_s_*_-falls_, *r_s_*_-suffocation_, *r_s_*_-MVT_, *r_s_*_-fire/burn_, *r_s_*_-natural_, and *r_s_*_-poisoning_: Spearman correlation coefficients between unspecified unintentional injury mortality and mortality rates from falls, motor vehicle traffic crashes, suffocations, fires/burns, natural/environmental causes, and poisonings, respectively.
